# A V_H_H-Fc Fusion Targeted to the Chloroplast Thylakoid Lumen Assembles and Neutralizes Enterohemorrhagic *E. coli* O157:H7

**DOI:** 10.3389/fpls.2021.686421

**Published:** 2021-05-28

**Authors:** Adam Chin-Fatt, Rima Menassa

**Affiliations:** ^1^Agriculture and Agri-Food Canada, London Research and Development Centre, London, ON, Canada; ^2^Department of Biology, University of Western Ontario, London, ON, Canada

**Keywords:** enterohemorrhagic *E. coli*-EHEC, IgA, single domain antibody, V_H_H, Fc fusion, Fc engineering, thylakoid, chloroplast

## Abstract

Chimeric fusion proteins comprising a single domain antibody (V_H_H) fused to a crystallizable fragment (Fc) of an immunoglobulin are modular glycoproteins that are becoming increasingly in demand because of their value as diagnostics, research reagents and passive immunization therapeutics. Because ER-associated degradation and misfolding may potentially be limiting factors in the oxidative folding of V_H_H-Fc fusion proteins in the ER, we sought to explore oxidative folding in an alternative sub-compartment, the chloroplast thylakoid lumen, and determine its viability in a molecular farming context. We developed a set of in-house expression vectors for transient transformation of *Nicotiana benthamiana* leaves that target a V_H_H-Fc to the thylakoid lumen via either secretory (Sec) or twin-arginine translocation (Tat) import pathways. Compared to stromal [6.63 ± 3.41 mg/kg fresh weight (FW)], cytoplasmic (undetectable) and Tat-import pathways (5.43 ± 2.41 mg/kg FW), the Sec-targeted V_H_H-Fc showed superior accumulation (30.56 ± 5.19 mg/kg FW), but was less than that of the ER (51.16 ± 9.11 mg/kg FW). Additionally, the introduction of a rationally designed *de novo* disulfide bond enhances *in planta* accumulation when introduced into the Sec-targeted Fc fusion protein from 50.24 ± 4.08 mg/kg FW to 110.90 ± 6.46 mg/kg FW. *In vitro* immunofluorescent labeling assays on V_H_H-Fc purified from Sec, Tat, and stromal pathways demonstrate that the antibody still retains V_H_H functionality in binding Escherichia *coli* O157:H7 and neutralizing its intimate adherence to human epithelial type 2 cells. These results overall provide a proof of concept that the oxidative folding environment of the thylakoid lumen may be a viable compartment for stably folding disulfide-containing recombinant V_H_H-Fc proteins.

## Introduction

Chimeric fusion proteins comprising a single domain antibody (V_H_H) fused to a crystallizable fragment (Fc) of an immunoglobulin A (IgA) are modular glycoproteins that are becoming increasingly in demand because of their potential value as passive enteromucosal immunization therapeutics (Harmsen and De Haard, 2007). Structurally, they differ from the native IgA monomer in that the antigen binding fragment (Fab) has been replaced by a camelid-derived V_H_H that is smaller (∼15 kDa), more stable and does not require multi-chain assembly as is the case for Fab heavy and light chains (De Meyer et al., 2014). We have previously produced a V_H_H-Fc in *Nicotiana benthamiana* leaf tissue by targeting it to the endoplasmic reticulum (ER) for folding and demonstrated its functionality *in vitro* in binding and neutralizing four serotypes of enterohemorrhagic Escherichia *coli* (EHEC), including O157:H7, the most prevalent serotype in North America (Saberianfar et al., 2019; Chin-Fatt et al., 2021). The national food surveillance programs for foodborne pathogens in the United States and Canada, FoodNet and FoodNet Canada, respectively, reported that the average incidence rate for O157:H7 was most recently estimated at 0.8 per 100,000 persons in the United States (CDC, 2020) and 1.52 per 100,000 persons in Canada (PHAC, 2019). Because the bovine terminal rectum is the primary reservoir of the pathogen, an appealing pre-harvest intervention strategy has been to encapsulate IgA in plant material as part of a feed formulation intended for enteromucosal passive immunization. Seeds of *Arabidopsis thaliana* encapsulating a V_H_H-Fc secretory complex have been demonstrated to be effective in passively immunizing weaned piglets against a related *E. coli* strain, enterotoxigenic *E. coli*, and in reducing bacterial shedding to beneath detection levels after 4 days (Virdi et al., 2013). Over the past 20 years, plants have become a platform of choice for producing IgA antibodies and related synthetics in part because of the requirement of disulfide bond formation for proper folding and assembly (Vasilev et al., 2016). In plant cells, the oxidative folding of native proteins with disulfide bonds is localized either in the ER, the mitochondrial intermembrane space or the chloroplast thylakoid lumen (Onda, 2013).

Chloroplasts are structurally complex organelles that consist of a double membrane enclosing a soluble stromal phase (Kirchhoff, 2019). Within the stroma, an independent membrane system known as the thylakoid is embedded with the chlorophyll-containing photosystems responsible for photosynthesis and further encloses an additional luminal phase (Pottosin and Shabala, 2016; Johnson and Wientjes, 2019). Protein transport across these membranes is differentially regulated and each of these compartments can be considered to have different proteomic profiles and contain different folding environments (Lee et al., 2017). Proteins that are encoded by the nucleus, synthesized in the cytosol and localized in the thylakoid lumen require an *N*-terminal bipartite transit peptide consisting of two signals in tandem: a signal to enter the TIC/TOC import system of the outer double membrane followed by a thylakoid targeting signal that uses one of two functionally distinct pathways, known as the secretory (Sec) and twin-arginine translocation (Tat) pathways (Pottosin and Shabala, 2016; Fernandez, 2018; Johnson and Wientjes, 2019; Palmer and Stansfeld, 2020). The Sec pathway bears homology to the bacterial secretory pathway and actively transports the unfolded pre-protein bound to the SecA chaperone through a Sec membrane complex (Ries et al., 2020). On the other hand, protein transport via the Tat pathway uses a transthylakoidal proton gradient as its energy source and can mediate transport of fully folded globular proteins across the thylakoid membrane (Palmer and Stansfeld, 2020). The Tat pathway is also unique in having a protein proofreading ability that targets misfolded proteins for degradation (Robinson et al., 2011). In the thylakoid lumen, a single chimeric protein, lumen thiol oxidoreductase I (LTO1), performs both *de novo* formation and transfer of disulfides to proteins that undergo oxidative folding (Karamoko et al., 2013). Folding of both the Fc and the V_H_H requires the formation of intra-chain disulfide bonds as a structure-stabilizing modification to prevent denaturation and reduce susceptibility to proteolysis (Woof and Russell, 2011; Vincke and Muyldermans, 2012). Structurally, the Fc chain consists of two distinct domains, CH2 and CH3, each comprising two anti-parallel beta sheets that are connected at the center by an intra-chain disulfide bond. The Fc also homodimerizes via inter-chain disulfide bonds that cross-link cysteines on the CH2 domain near the hinge. Similarly, the V_H_H is predicted to typically contain one to two intra-chain disulfides (Wesolowski et al., 2009), and when produced in bacteria, requires targeting to the periplasm for correct folding (Henry et al., 2017). Because the Sec and Tat pathways differ in trafficking the unfolded and folded protein cargo, respectively, we hypothesized that the inter-chain and intra-chain disulfide formation of the V_H_H-Fc would be exclusive to the Sec pathway and may be evident in its dimerization and stable accumulation, respectively.

The oxidizing environment of the thylakoid lumen has conventionally been considered the only site for oxidatively folding chloroplast proteins that require disulfide stabilization because of the control of a *trans*-thylakoid redox pathway (Karamoko et al., 2013). However, a few studies have suggested that proteins requiring disulfide formation may unexpectedly fold and be biologically active in the reducing environment of the stroma (Staub et al., 2000; Daniell et al., 2001; Mayfield et al., 2003; Bally et al., 2008). These discrepancies may possibly be explained by spontaneous disulfide formation in the stroma or activity by the membrane embedded LTO1 even though previous studies have suggested it to be oriented toward the interior of the lumen (Kieselbach and Schroder, 2003; Lu et al., 2013). To differentiate between the ability of the stroma and thylakoid lumen to properly introduce disulfide bonds and fold a V_H_H-Fc protein, we targeted it to both compartments.

In this study, we have compared accumulation levels of a V_H_H-Fc that was transiently targeted to the chloroplast thylakoid compartment via Sec and Tat pathways or to the chloroplast stroma in *N. benthamiana* leaf cells. We have also demonstrated that the V_H_H-Fc targeted to the thylakoid or stromal compartments is functional in binding and neutralizing adherence of *E. coli* O157:H7 to human epithelial cells. This study is notable because it provides a proof of concept that the folding environment of the thylakoid lumen is conducive for accumulating a V_H_H-Fc fusion that retains functionality in binding and neutralizing its target, and provides a foundation for producing transplastomic plants targeting such an antibody for folding and accumulation in the thylakoid lumen.

## Materials and Methods

### Cloning and Expression

**FIGURE 1:**
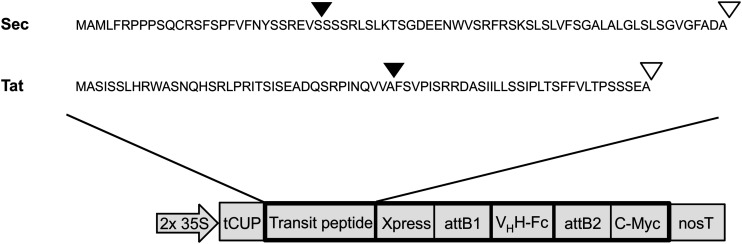
Construction of thylakoid expression vectors. Transit peptide sequences used for thylakoid targeting via either Sec or Tat pathways. Triangles indicate predicted cleavage sites after entry into stromal (black triangles) and thylakoid (white triangles) compartments. 2 × 35S: double-enhanced promoter from Cauliflower Mosaic Virus 35S gene; tCUP, translational enhancer from a tobacco cryptic upstream promoter; attB1/attB2, cloning sites used for Gateway cloning; nosT, nopaline synthase transcription terminator; and Xpress/c-myc, detection/purification tags.

Both Sec and Tat sequences (Accession #s: NP_001318791 and NP_001321139, respectively) were obtained from a previous proteomics study that isolated and sequenced multiple luminal proteins in *A. thaliana* (Schubert et al., 2002). The Tat targeting sequence corresponds to the *N*-terminal 71 amino acids of a FKBP-type peptidyl-prolyl *cis-trans* isomerase (At1g20810). The Sec targeting sequence corresponds to the *N*-terminal 75 amino acids of a thylakoid luminal 15.0 kDa protein 2 (At5g52970; Figure 1). Cleavage sites of the targeting peptides were predicted using the ChloroP and TargetP online tools (Emanuelsson et al., 1999; Almagro Armenteros et al., 2019). Sequences were synthesized by BioBasic Inc. (Mississauga, Canada) and then cloned using a sequence and ligation independent cloning method (SLIC; Li and Elledge, 2007) into an in-house developed cytosolic expression vector, pCaMGate (Pereira et al., 2014). The pCaMGate vector attaches an *N*-terminal Xpress tag for protein stability and a *C*-terminal c-myc tag for detection and purification. The V_H_H-Fc construct was developed previously and consists of an anti-EHEC V_H_H fused to a bovine Fc (ANN46383; described as V_H_H9; Saberianfar et al., 2019). The V_H_H-Fc was cloned into this adapted vector by Gateway^®^ cloning and the reading frame was confirmed by sequencing. For stromal and ER expression, we used a set of previously developed in-house expression vectors termed “pCaMGate stroma” and “pCaMGate ER,” respectively (Pereira et al., 2014). pCaMgate stroma attaches an *N*-terminal transit peptide derived from the small subunit of RuBisCO. The ER vector attaches an *N*-terminal PR1b tobacco signal peptide for targeting to the secretory pathway and a *C*-terminal KDEL tag for retrieval to the ER. Aside from the signal peptides, the sequences of the stroma and ER expression vectors are identical to the Sec and Tat expression vectors.

For transient expression in plants, expression vectors were transformed by electroporation into *Agrobacterium tumefaciens* (EHA 105) and correct clones selected using 50 μg/ml kanamycin and 10 μg/ml rifampicin antibiotics. Starter cultures of Luria Bertani (LB) medium were inoculated with individual colonies and grown overnight with kanamycin and rifampicin. A starter culture with p19, a suppressor of post-transcriptional silencing (Silhavy et al., 2002), was also grown overnight. The starter culture was then diluted 1:1000 into an infiltration culture consisting of LB medium, 50 μg/ml kanamycin, 10 μg/ml rifampicin, 10 mM 2-(*N*-morpholino)ethanesulfonic acid (MES) at pH 5.6 and 100 μM acetosyringone. Cultures were incubated overnight at 28°C with 250 rpm shaking until an optical density at 600 nm (OD600) of 0.5 was reached using a Nanodrop 2000c spectrophotometer (Thermo-Fisher Scientific, Cat. No. ND-2000c). Cultures were then centrifuged at 4,000 × *g* for 30 min and the pellet resuspended to an OD600 of 1.0 in Gamborg’s solution containing 3.2 g/l Gamborg’s B5 with vitamins, 20 g/l sucrose, 10 mM MES (pH 5.6), and 100 μM acetosyringone. Cultures were then incubated at room temperature using an end over end rotator for 1 h. Equal volumes of the culture with V_H_H-Fc, culture with p19 and Gamborg’s solution were then combined and used to infiltrate leaves of 8 to 10 week old *N. benthamiana* plants. Plants were grown in a growth cabinet with a 16 h light/8 h dark cycle, maintained at 21–22°C, with 55% humidity and exposed to 100 μmol/photons m^–2^ s^–1^ of light during day cycles. Agroinfiltration involved puncture of the basal leaf surface using a needle followed by injection of the culture into the leaf using a 3 mL syringe. Following agroinfiltration, plants were returned to the growth cabinet before sampling leaf tissue.

### Visualizing Subcellular Compartment Localization

To visualize subcellular compartment localization, GFP was cloned into the 3’ end of the V_H_H-Fc sequence in a pUC57 vector by SLIC and the reading frame was confirmed by sequencing. The V_H_H-Fc-GFP construct was then cloned into Sec, Tat, and chloroplast stroma expression vectors by Gateway cloning and each was agroinfiltrated into leaves of *N. benthamiana*. Tissue was harvested after 2 days, mounted in Aqua-Poly/Mount (Polyscience Inc., Warrington, PA, United States, Cat. No. 18606) and visualized using a 60 × water immersion objective lens and an Olympus LSM FV 1200 confocal microscope for Sec and stroma samples or a Leica TCS SP2 CLSM confocal microscope for Tat samples. Samples were excited at 488 nm using a multi-argon laser set at 5% and emission was detected at 500–545 nm for GFP and at 630–690 nm for chlorophyll.

### Protein Extraction and Western Blot

Pre-weighed leaf samples were frozen in liquid nitrogen and homogenized with silica beads (Bio Spec Products Inc., Bartlesville, OK, United States) for 2 min using a TissueLyser II (Retsch Inc., Newton, PA, United States). One milliliter of a denaturing extraction buffer [1× PBS, pH 7.5, 4% sodium dodecyl sulfate (SDS), 2% polyvinylpolypyrrolidone (PVPP)] was added per approximately one hundred milligrams of sample. All samples were then vortexed on high speed for 30 s and centrifuged at 20,000 × *g* for 10 min to remove cell debris. Extracted protein samples were combined with either a 5 × reducing loading buffer [0.3 M Tris–HCl pH 8.0, 5% SDS, 10% glycerol, 100 mM Dithiothreitol (DTT), 0.05% Phenol Red] or a 5 × non-reducing buffer (0.3 M Tris–HCl pH 8.0, 5% SDS, 10% glycerol, and 0.05% Phenol Red), heated at 90°C for 10 min, then loaded onto Express Plus PAGE 4–20% gradient gels (Genscript Inc., Piscataway, NJ, United States). Gels were run at 100 V for 100 min, then transferred to polyvinylidine difluoride membrane using the *Trans*-Blot Turbo transfer system (Bio-Rad Laboratories Inc., Hercules, CA, United States). Blots were blocked overnight with 5% (w/v) skimmed milk in 1× tris-buffered saline with 0.1% tween-20 (TBS-T), pH 7.5, and proteins of interest were probed with a mouse anti-c-myc antibody (diluted 1:5,000; Genscript Inc., Piscataway, NJ, United States) and the 1-H Basic Western kit for mouse primary antibody (Genscript Inc., Piscataway, NJ, United States). Membranes were washed three times in TBS-T for 10 min each and then incubated for 5 min with either Amersham ECL Western Blot detection reagents (GE Healthcare, Mississauga, ON, Canada) or Enhanced Chemiluminescent detection solution (Biorad Laboratories Inc., Hercules, CA, United States). Membranes were placed into a plastic cover to prevent desiccation and were imaged using a MicroChemi 4.2 imaging system with GelCapture acquisition software (DNA Bio-Imaging Systems Ltd., Jerusalem, Israel). Quantification of accumulation was done using densitometry and a calibrated standard curve based on an in-house developed protein of ∼55 kDa of known concentration using the Totallab TL100 software (Non-linear Dynamics, Durham, United Kingdom). Statistical significance was determined using a two-tailed unpaired *T*-test on 3–5 biological replicates.

### Recombinant Protein Purification

Leaf tissue was extracted in approximately one mL of a mild native extraction buffer (1× PBS, pH 7.5, 0.1% Tween-20, 1 mM EDTA, 2% PVPP, 100 mM sodium ascorbate, 8 M sucrose, 1 μg/mL leupeptin, 1 mM PMSF, and 1 μg/mL pepstatin A) per one hundred mg of tissue. Samples were vortexed and the supernatant collected after two rounds of centrifugation at 22,000 × *g* for 20 min each. The recombinant protein in the clarified extract was then purified by affinity chromatography using an anti-c-myc purification kit (MBL International Corp., Woburn, MA, United States) according to the manufacturer’s protocol. Briefly, 100 μL anti-c-Myc tag bead suspension was added to 3 mL of clarified extract and incubated in a 4°C room for 1 h using an end-over-end shaker to hybridize the beads to the *C*-terminal c-myc tag on the V_H_H-Fc protein. The extract was then transferred to a spin column and centrifuged for 10 s. The beads were then washed three times with the provided washing solution and the protein was eluted by competition using a c-Myc tag peptide in 1x PBS at neutral pH.

### *E. coli* O157:H7 Binding Assay

Escherichia *coli* strain O157:H7 was obtained from Dr. Michael Mulvey (Public Health Agency of Canada, National Microbiology Laboratory, *E. coli* Unit, Enteric Diseases Program, Winnipeg, MB, Canada) and stored at -80°C in a level 2 containment laboratory. Binding assays were performed as previously described (Saberianfar et al., 2019). A summary is as follows. A 3 mL culture of *E. coli* O157:H7 was grown overnight in LB medium at 37°C. The culture was then pelleted, rinsed repeatedly in 1× PBS, then fixed in 4% paraformaldehyde. The fixed cells were incubated with 2 μg purified V_H_H-Fc for 1 h at 37°C. The cells were hybridized to a FITC-conjugated secondary antibody (rabbit anti-bovine-FITC; 1:40 dilution, Thermo-Fisher Scientific, Cat. No. SA1-36043) that binds Fc. To stain the bacteria, the cells were briefly incubated with DAPI and then washed with 1x PBS. The cells were then dried onto poly-L-lysine coated coverslips (Millipore Sigma, Cat, No. S1815) and mounted onto glass slides with Aqua-Poly/Mount (Polyscience Inc., Warrington, PA, United States, Cat. No. 18606). FITC and DAPI sequential imaging was performed by confocal microscopy with a 64× water lens and an Olympus LSM FV 1200 confocal microscope. FITC was imaged by excitation with a 480 nm laser and detection at 520–540 nm. DAPI was imaged by excitation at 350 nm and detection at 455–465 nm.

### HEp-2 Adherence Inhibition Assay

Inhibition assays were performed as previously described (Saberianfar et al., 2019). A summary is as follows. HEp-2 cells (ATCC) were grown to ∼80% confluency and used to seed eight-well chamber slides overnight with ∼2 × 10^5^ cells per well in pre-warmed Dulbecco’s Modified Eagle Medium (DMEM; Thermo-Fisher Scientific, Cat. No. 10566016) supplemented with 10% fetal bovine serum (FBS) at 37°C in 5% CO_2_. At the start of the assay, the overnight medium was replaced with 225 μL fresh DMEM. *E. coli* strain O157:H7 was grown overnight in LB medium and subcultured to a 1:50 dilution in pre-warmed DMEM with and without 2 μg V_H_H-Fc and then incubated with the HEp-2 cells at 37°C in 5% CO_2_ for 3 h without shaking. The cultures were washed with 1× PBS to remove non-adherent bacteria, fixed in 4% paraformaldehyde, washed repeatedly again with 1× PBS, and hybridized with Alexa 647 phalloidin (Thermo-Fisher Scientific, Cat. No. A22287) to visualize actin in the HEp-2 cells, and donkey anti-rabbit Alexa 350 (Thermo Fisher Scientific Cat. No.A10039) to visualize O157:H7 cells. To visualize adherence to HEp-2 cells, sequential imaging was performed using an Olympus LSM FV 1200 confocal microscope with a 64x water objective lens. Alexa 647 phalloidin was imaged by excitation at 650 nm and detection at 660–680 nm. The donkey anti-rabbit Alexa 350 antibody was visualized by excitation at 350 nm and detection at 455–465 nm.

## Results

### Sub-Compartment Targeting Influences Accumulation and Dimerization Patterns of the V_H_H-Fc Fusion

**FIGURE 2:**
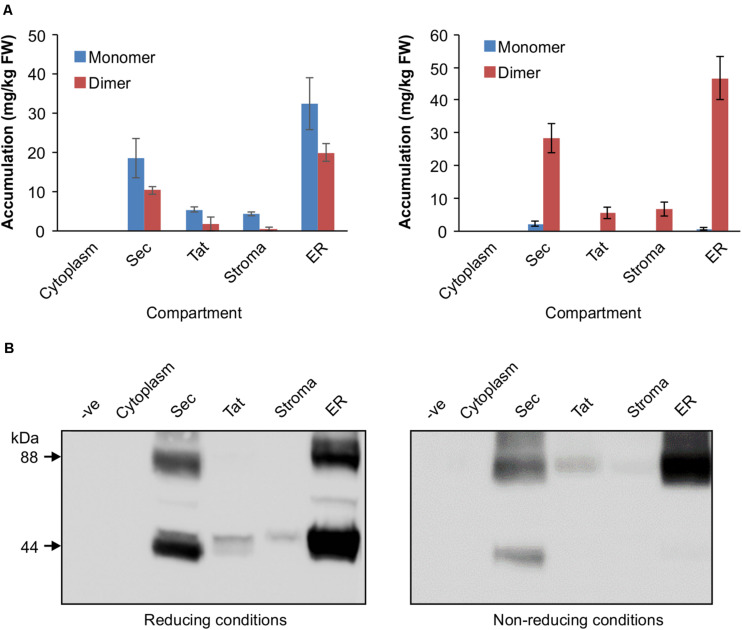
Accumulation profiles of V_H_H-Fc targeted with cytoplasm, Sec, Tat, stromal, and ER signals. **(A)** Bar chart comparing V_H_H-Fc accumulation levels across cytoplasm, Sec, Tat, stromal, and ER signals extracted in reducing (left) or non-reducing (right) conditions. *N* = 3 biological replicates. Error bars shown are standard error. **(B)** Representative Western blot showing relative accumulation of V_H_H-Fc across compartments in reducing (left) and non-reducing (right) conditions.

The V_H_H-Fc was cloned into five separate plant expression vectors that permit targeting of the protein to the chloroplast thylakoid via Sec or Tat pathways, the chloroplast stroma, the ER or the cytosol. After transiently transforming leaves of *N. benthamiana*, tissue was harvested and crude extract separated by SDS-PAGE in either a reducing buffer or a non-reducing buffer to distinguish disulfide-based dimerization of the V_H_H-Fc. We did not observe a difference in chlorosis or necrosis of the areas infiltrated with the various constructs compared to the negative control (p19). Although there are no crystal structures for bovine IgA, X-ray crystal structures for human IgA (Herr et al., 2003; Ramsland et al., 2007), which bears a 70% sequence similarity, suggest that three, or possibly four, cysteines on each CH2 domain of the Fc will form disulfide linkages. Under non-reducing conditions, which retain disulfide bonds in the protein, the V_H_H-Fc is detected predominantly as an 88 kDa band matching the predicted size of the V_H_H-Fc dimer. Total accumulation is highest in the ER at 51.16 ± 9.11 mg/kg FW, followed by the thylakoid via Sec-targeting at 30.56 ± 5.19 mg/kg FW. Accumulation in the stroma and thylakoid via Tat-targeting are substantially lower at 6.63 ± 3.41 mg/kg FW and 5.43 ± 2.41 mg/kg FW, respectively, and is undetectable in the cytosol. Under reducing conditions, the same samples display an enriched band at 44 kDa matching the predicted size of the V_H_H-Fc monomer for the ER, stroma, thylakoid via Sec and via Tat suggesting that the V_H_H-Fc dimer in these compartments is stabilized by an inter-chain disulfide bond (Figures 2A,B).

### Sec- and Tat-Targeted Fc-GFP Localizes in the Thylakoid

**FIGURE 3:**
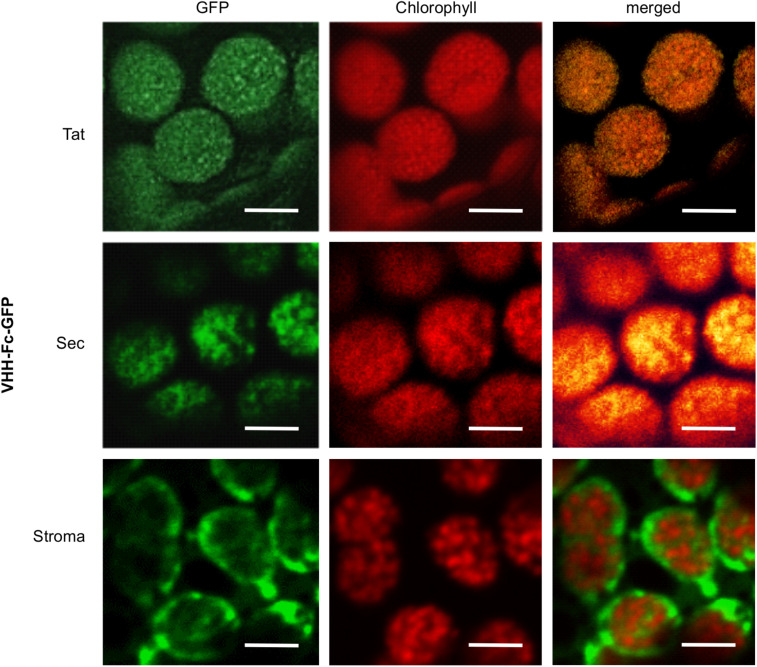
Sec and Tat transit peptides enable localization of the recombinant Fc-GFP to the thylakoid chloroplast sub-compartment. Confocal images visualizing GFP-tagged Fc (green) targeted to the chloroplasts with either Sec, Tat or stromal signals. Chlorophyll (red) indicates the locations of the thylakoid grana. Fluorescence was sequentially captured, and the merged images show co-localization of GFP and chlorophyll (yellow). Size bar = 10 μm.

To verify that the Tat and Sec transit peptides indeed target the V_H_H-Fc to the chloroplast thylakoid sub-compartment, we tracked subcellular localization of the Fc by fusing GFP to the Fc chain in each of the expression vectors. Visualization by confocal microscopy showed the Sec and Tat-targeted GFP-tagged protein to consistently co-localize with chlorophyll, which accumulates in the thylakoid and auto-fluoresces at ∼735 nm (Figure 3). On the other hand, the construct targeting the recombinant protein to the stroma showed a very distinct pattern surrounding the thylakoid grana, and into stromules. Therefore, the Sec and Tat transit peptides we used indeed target the recombinant protein to the thylakoid.

### Sec-Targeted V_H_H-Fc Fusions With an Engineered Disulfide Show Improved Yield

**FIGURE 4:**
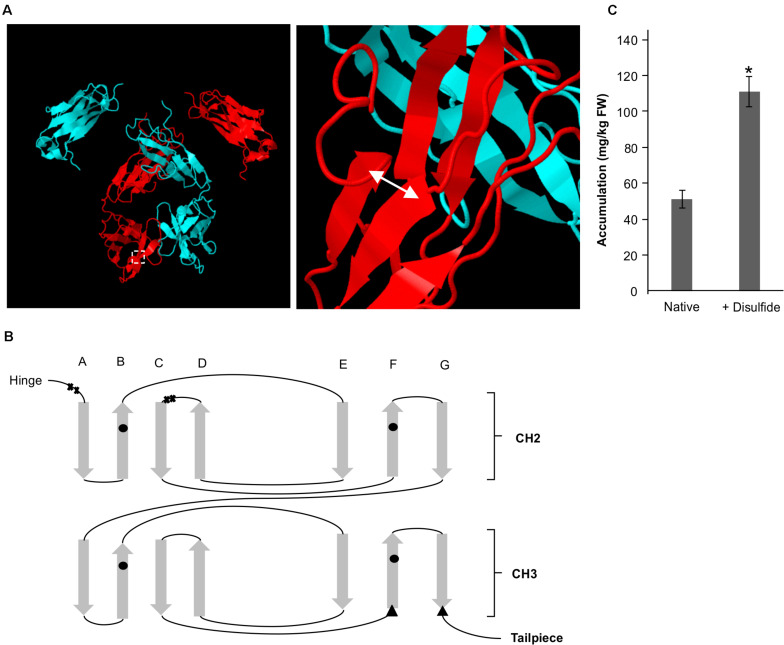
A *de novo* disulfide bond enhances accumulation for a Sec-targeted V_H_H-Fc. **(A)** Structural model showing the backbone of two dimerized Fc chains (blue and red). The double headed arrow indicates the position of the introduced disulfide. **(B)** Greek key connectivity schematic showing the relative positions of native disulfides and the introduced disulfide in the Fc. X indicates cysteines involved in interchain disulfide formation, circles indicate cysteines involved in intrachain disulfide formation and triangles indicate introduced cysteines for *de novo* disulfide formation. **(C)** Bar chart showing accumulation of Sec-targeted native V_H_H-Fc and a V_H_H-Fc with an added disulfide. *indicates statistical significance as determined by a *T*-test with *p* < 0.05, *n* = 3 biological replicates. Error bars shown are standard error of the mean.

We have previously identified by rational design a residue pair (G196C/R219C) on the Fc that if mutated to cysteines would form a *de novo* disulfide bond that enables a ten-fold improvement in yield of the V_H_H-Fc when targeted to the ER (Chin-Fatt et al., 2021). The pair forms an intra-chain disulfide between strand G and strand F on the CH3 domain in oxidative folding conditions (Figures 4A,B). To determine if the oxidative folding of the thylakoid can recapitulate the yield-improving effects of an engineered disulfide bond, we targeted the V_H_H-Fc fusion carrying the G196C/R219C mutation to the thylakoid lumen via the Sec pathway and measured accumulation by western blotting after agroinfiltration. The engineered V_H_H-Fc showed a significant yield improvement at 110.90 ± 6.46 mg/kg FW compared to the native V_H_H-Fc at 50.24 ± 4.09 mg/kg FW (Figure 4C), suggestive of the ability of the thylakoid lumen to incorporate *de novo* disulfide bonds on a heterologous V_H_H-Fc via the Sec pathway.

### Sec, Tat, and Stroma-Targeted V_H_H-Fc Fusions Bind O157:H7

**FIGURE 5:**
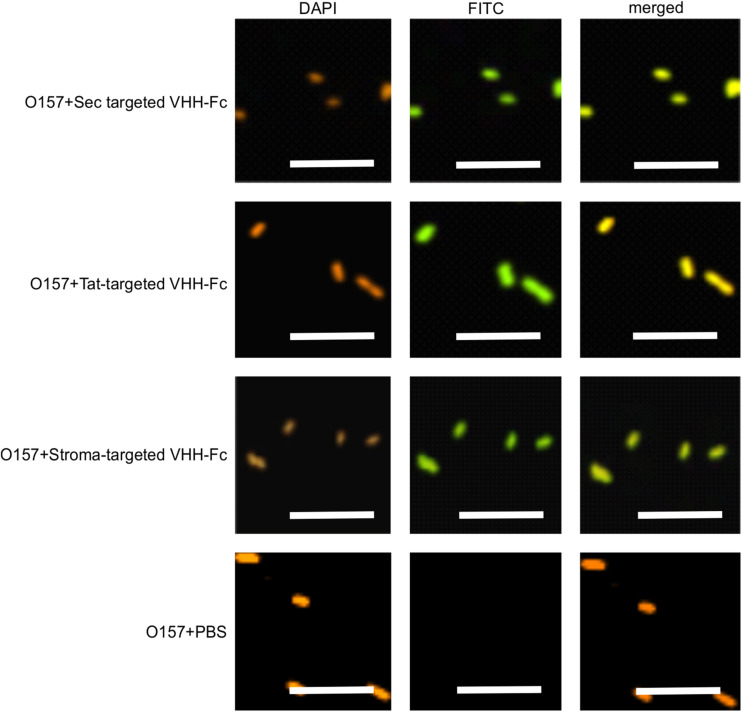
V_H_H-Fc targeted with either Sec, Tat or stromal transit peptides can bind O157:H7 cells. Shown are confocal images of V_H_H-Fc targeted with either Sec, Tat or stromal signals incubated with EHEC O157:H7. DAPI was used to visualize EHEC cells (orange) and a FITC-conjugated antibody (green) was used to immunolabel the Fc specifically. Size bar = 10 μm.

We previously demonstrated that the ER-targeted V_H_H binds to intimin, an integral outer membrane protein of *E. coli* O157:H7 (Saberianfar et al., 2019). To determine if the thylakoid-targeted V_H_H-Fc retained the ability to bind *E. coli* O157:H7, purified V_H_H-Fc from each compartment was incubated with the pathogen then fixed in paraformaldehyde, washed and probed for immunofluorescence using a FITC labeled anti-c-myc secondary antibody. Visualization by confocal microscopy showed consistent co-localization between DAPI-stained bacterial cells and the FITC-labeled V_H_H-Fc for the thylakoid via Sec, thylakoid via Tat, and stromal compartments, indicating that the chloroplast-targeted V_H_H-Fc retains the ability to bind intimin on EHEC surfaces (Figure 5). As a negative control, O157:H7 cells were also treated with 1x PBS containing 0.1% Tween-20 (PBS-T) instead of the V_H_H-Fc and similarly stained but did not show fluorescence under FITC-related imaging conditions (480 nm excitation and 520–540 nm detection). This result suggests that the V_H_H is folded properly and recognizes its target when Sec-, Tat-, and stroma-targeted.

### Sec-, Tat- and Stroma-Targeted V_H_H-Fc Fusions Can Neutralize O157:H7’s Adherence to HEp-2 Cells

**FIGURE 6:**
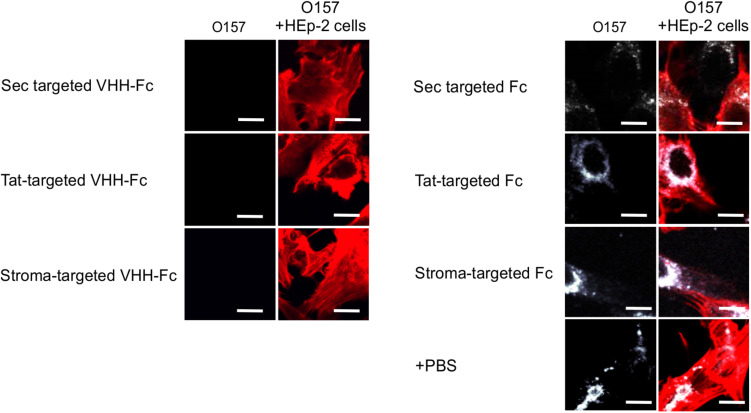
V_H_H-Fc targeted with either Sec, Tat or stromal transit peptides can neutralize adherence of O157:H7 to HEp-2 cells. Shown are confocal images of O157:H7 (white) that has been incubated with Hep-2 cells (red) in the presence of either V_H_H-Fc targeted to Sec, Tat, and stromal compartments or Fc as a negative control targeted to the same compartments. Size bar = 20 μm.

Given that intimin mediates the attachment of *E. coli* O157:H7 to intestinal epithelial cells, and that V_H_H-Fc targeted to the ER neutralizes *E. coli*’s ability to adhere to those cells (Chin-Fatt et al., 2021), we tested if thylakoid targeting of the V_H_H-Fc impacted its ability to neutralize the bacterium from adhering to epithelial cells by blocking intimin. HEp-2 cells were incubated with *E. coli* O157:H7 in the presence or absence of purified V_H_H-Fc from each of the compartments. Cells were then washed to remove non-adherent bacteria, fixed in paraformaldehyde, and incubated with immunofluorescent labels. Human epithelial type-2 (HEp-2) cells were visualized by fluorescent actin staining using rhodamine phalloidin (shown in red) and O157:H7 cells visualized using a donkey anti-rabbit alexa 350 secondary antibody (shown in white; Figure 6). Compared to the control lacking V_H_H-Fc, and to the control Fc lacking the V_H_H, the addition of V_H_H-Fc from any of the compartments seems to abrogate adhesion of any labeled *E. coli* O157:H7 to the incubated HEp-2 cells as visualized using confocal microscopy (Figure 6). This indicates that the chloroplast-targeted V_H_H-Fc retains the ability to neutralize EHEC from colonizing epithelial cells and that the inhibition of adhesion is mediated by the V_H_H and not by non-specific interactions of the Fc moiety of the antibody.

## Discussion

### Utility of a Thylakoid Targeting System for Antibodies

The utility of a plant platform for folding and assembling IgA antibodies and related synthetics in the ER is well established (Ma et al., 1998; Nakanishi et al., 2013; Virdi et al., 2013; Saberianfar et al., 2019). However, in a previous study exploring how recombinant antibodies influence the endogenous proteome, a genome-wide Tiling array suggested that ER-targeted V_H_H-IgG Fc fusions in *A. thaliana* seeds generated an unfolded protein response (De Wilde et al., 2013). Because the thylakoid has a different proof-reading system for folding than the ER, there may be value in exploring it as an alternative oxidative folding compartment for antibody folding because of the potential to avoid ER-associated degradation as a limiting factor. In this study, we explored the possibility of producing a V_H_H-Fc fusion in the thylakoid lumen as a viable yield-optimization strategy within the context of molecular farming. Overall, the results suggest that the V_H_H-Fc fusion seems to fold correctly and assemble with the requisite intra- and inter-chain disulfides as well as retains binding and neutralization efficacy. Although accumulation was not found to be higher than the ER, purified V_H_H-Fc protein from both stromal and luminal fractions retained binding and neutralization efficacy. Notably, the Sec-targeted V_H_H-Fc fusion accumulated significantly better than stromal and Tat-targeted fusions, albeit at approximately 60% of the ER-targeted V_H_H-Fc fusion. Additionally, the Sec-targeted V_H_H-Fc was detected as two bands in non-reducing conditions, corresponding to monomeric and dimeric forms, while the ER-targeted V_H_H-Fc was detected as a single band corresponding to the dimer (Figure 2B). This may indicate either relatively slower or less efficient interchain disulfide formation with Sec-targeted V_H_H-Fc compared to the ER, or alternatively, that there is detection of the unfolded preprotein before thylakoid import. Nonetheless, previous experience has suggested that robust accumulation for a transiently expressed, chloroplast targeted recombinant protein is indicative of high yields upon developing a stable transplastomic line for that protein (Kolotilin et al., 2012). Transplastomically-expressed recombinant proteins tend to be of higher yield than when nuclear-expressed due to the polyploidy of the chloroplast genome and the lack of silencing and positional interaction effects (Bock, 2007; Daniell et al., 2009). The use of stably-transformed chloroplasts presents several unique advantages as a molecular farming strategy, notably maternal inheritance of the chloroplast genome which virtually eliminates the prospect of gene escape to the environment by pollen (Kumar et al., 2004). Additionally, the recombinant proteins are encapsulated by chloroplast membranes and are effectively isolated from cellular proteases which are more abundant and diverse than those found in the chloroplast. The proteome and protease profile of the thylakoid lumen in particular is substantially more limited in comparison (Kieselbach and Schroder, 2003). Chloroplast-based expression might also facilitate alternative purification methods because intact chloroplasts can be easily isolated from crude extracts by low-speed centrifugation (Kubis et al., 2008). If yields are high enough, there may be value in scaling up production of biomass that could then be administered orally to animals for enteric protection against EHEC without the need for purification.

### Disulfide Formation in the Chloroplast

**FIGURE 7:**
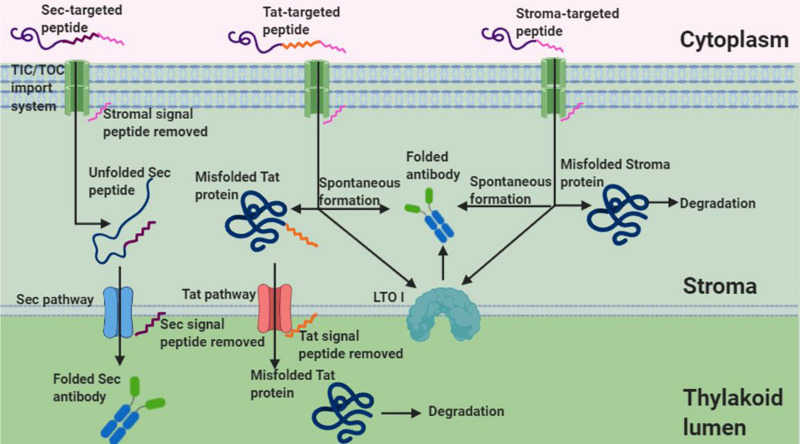
Schematic of Sec, Tat, and stromal import of an antibody. Given that Tat and stromal imported antibodies retain functionality and show dimerized banding under non-reducing conditions, this suggests disulfide formation in the stroma despite its reducing environment. Shown are two possible mechanisms of disulfide formation, interaction with LTOI and spontaneous formation, that may account for disulfide formation of tat and stroma targeted antibodies (Figure created in Biorender.com).

Under non-reducing conditions, banding corresponding to the V_H_H-Fc dimer was unexpectedly detected for the stromal and Tat-targeted compartments. Disulfide formation has conventionally been thought to be exclusive to the oxidative folding environment of the thylakoid. However, a few studies have suggested that disulfide formation is possible in the reducing environment of the stroma for recombinant proteins, though it tends to be at much lower levels and the mechanics of which remain uncertain. For example, human growth hormone (Staub et al., 2000), cholera toxin B (Daniell et al., 2001), a recombinant alkaline phosphatase A (Bally et al., 2008), aprotinin (Tissot et al., 2008), and zeolin (De Marchis et al., 2011) all require disulfide bond formation for folding and are nonetheless biologically active when either expressed in or targeted to the stroma. Notably, Bally et al. (2008) have shown not only that the chloroplast stroma supports the formation of an active alkaline phosphatase A enzyme but also that sorting of the alkaline phosphatase to the thylakoid lumen leads to larger amounts and more active enzyme. If accumulation can be assumed to be a correlative measure of how well folded a protein is, then the higher accumulation observed for the Sec-targeted V_H_H-Fc suggests that the oxidative folding environment in the thylakoid lumen is conducive for proper folding of the V_H_H-Fc with the requisite disulfide stabilization. In contrast, both the stromal targeted and Tat-targeted V_H_H-Fc have low accumulation levels and may be due to the suboptimal folding environment of the stroma. Similarly, the lack of detectable signal in the cytoplasm may be due to the inability of the V_H_H-Fc to fold sufficiently, particularly because the intra-chain disulfides are needed to stabilize the characteristic beta sandwich CH domains (Kumar et al., 2020). Several others have reported successfully expressing a V_H_H in the chloroplast stroma. A nuclear-expressed and stroma-targeted V_H_H was shown to be effective in potato plants in modulating enzyme function endogenously, albeit at a very low accumulation of 0.03% TSP (Jobling et al., 2003). Similarly, a transplastomically produced stromal V_H_H in tobacco retained binding efficacy against albumin lysozyme, a causative agent in proteinuria, but was produced at levels too low to be quantified and caused a semi-lethal pale-green seedling phenotype (Magee et al., 2004). Upon exposure to light, the stroma exhibits changes in its pH and redox state and consequently, its capacity for oxidative folding. The stroma has a neutral pH close to 7 under dark conditions but alkalizes to pH 8 upon illumination due to H^+^ being actively pumped into the thylakoid lumen (Werdan et al., 1975). Additionally, during photosynthesis, the NADPH/NADP^+^ ratio increases in the stroma causing a more reducing environment (Heineke et al., 1991). Conversely, the thylakoid lumen is acidified upon light exposure with a change in pH from 7.5 under dark conditions to 5.7 under saturating light (Takizawa et al., 2007). Notably, photosynthesis is known to modulate the activity of several enzymes in the stroma by reducing key disulfide bonds via a cascade involving ferredoxin, ferredoxin-thioredoxin reductase, and thioredoxins *m* and *f* (Schürmann and Buchanan, 2008). Prior to triggering of the cascade, the disulfides of these enzymes are at least partially oxidized despite the reducing environment of the stroma. Thus, the relatively low accumulation levels for the stroma- and Tat- targeted V_H_H-Fc may potentially be due to an environment made more reducing from photosynthetic activity. Future studies using these sorting signals may want to consider growing infiltrated plants in low light or extended dark conditions for optimal accumulation. On the other hand, V_H_Hs produced in the chloroplast of the green algae *Chlamydomonas reinhardtii* are competent in binding botulinum neurotoxin and were shown to accumulate to 5% TSP suggesting that there may be key plant-specific physiological factors that limit production in plant chloroplasts versus *C. reinhardtii* chloroplasts (Barrera et al., 2015). Indeed, the protein disulfide isomerase-like RB60 is partitioned between stroma and thylakoids in *C. reinhardtii* chloroplasts and has been suggested to potentially interface bidirectionally (Trebitsh et al., 2001). Conversely, lumen thiol oxidoreductase1 (LTO1), that catalyzes disulfide bond formation, is embedded in the thylakoid membrane of plants and is known to be preferentially oriented toward the thylakoid lumen (Karamoko et al., 2013). It may thus also be possible that aside from spontaneous disulfide formation that the low levels of V_H_H-Fc when stromal-targeted and Tat-targeted may be due to trace disulfide isomerase activity at this partition. Therefore, this suggests that the stability, and accumulation thereof, of the V_H_H-Fc may be a function of its redox potential as it relates to the reactivity of its cysteine residues’ thiol groups and/or the availability of disulfide isomerase activity (Figure 7).

We also introduced a *de novo* disulfide pair G196C/R219C into the V_H_H-Fc targeted with the Sec signal and found a significant yield improvement. Compared to the ten-fold yield improvement observed previously when targeted to the ER (Chin-Fatt et al., 2021), the yield improvement is substantially less at only about a two-fold improvement. This may possibly be due to the availability of relevant chaperones across the two compartments. The thylakoid lumen is known to contain a unique chaperonin cpn60/cpn10 system distinct from the stromal chaperonin system or the HSP family in the ER (Schlicher and Soll, 1996). Alternatively, the difference in yield improvement may be due to different activities of LTO1 in the thylakoid lumen versus the protein disulfide isomerase (PDI)-mediated folding reactions in the ER.

### Structural Considerations for Antibodies Targeted to the Thylakoid Lumen

Although the thylakoid lumen is capable of disulfide formation, it lacks the machinery for glycosylation. Given that V_H_Hs are not natively glycosylated by their host camelids, and are also competent when produced in *E. coli*, we hypothesized that the V_H_H would retain functionality when folded in the thylakoid (Herrmann et al., 2009). Additionally, V_H_Hs have been shown to be effective in neutralizing a broad array of other enteric pathogens (Vega et al., 2013; Shkoporov et al., 2015; Schmidt et al., 2016; King et al., 2018). Accordingly, the binding and neutralizing assays suggest that efficacy is retained despite the lack of glycosylation. Although the V_H_H alone may be sufficient for neutralization, yields are usually low and attaching the Fc has been shown to improve accumulation (Virdi et al., 2019). The Fc also enables improved avidity via its ability to multimerize thereby mediating agglutination. In enteromucosal conditions, neutralization of a pathogen’s ability to colonize epithelial cells is predominantly by steric hindrance via agglutination, known as immune exclusion (Li et al., 2020). Although the bovine IgA Fc is natively glycosylated, a recent study that characterized glycosylation on a plant-made ER- and apoplast-targeted Fc fusion protein demonstrated that preventing glycan attachment did not prevent the Fc from correctly folding (Xiong et al., 2019). Therefore, the thylakoid may be a suitable compartment for folding and accumulating functional V_H_H-Fc fusion proteins despite the lack of glycosylation.

Overall, this study provides a proof of concept that targeting to the thylakoid lumen via the Sec pathway allows for accumulation of a functional V_H_H-Fc fusion and may thus be a strategic way of producing these therapeutics while accruing the benefits of plastidial encapsulation.

## Data Availability Statement

The original contributions presented in the study are included in the article/supplementary material, further inquiries can be directed to the corresponding author/s.

## Author Contributions

AC-F and RM conceived the study. AC-F performed the experiments and wrote the manuscript. AC-F and RM edited the manuscript. Both authors contributed to the article and approved the submitted version.

## Conflict of Interest

The authors declare that the research was conducted in the absence of any commercial or financial relationships that could be construed as a potential conflict of interest.

## References

[B1] Almagro Armenteros J. J. Salvatore M. Emanuelsson O. Winther O. Von Heijne G. Elofsson A. (2019). Detecting sequence signals in targeting peptides using deep learning. *Life Sci. Alliance* 2:e201900429. 10.26508/lsa.201900429 31570514 PMC6769257

[B2] Bally J. Paget E. Droux M. Job C. Job D. Dubald M. (2008). Both the stroma and thylakoid lumen of tobacco chloroplasts are competent for the formation of disulphide bonds in recombinant proteins. *Plant Biotechnol. J.* 6 46–61. 17944820 10.1111/j.1467-7652.2007.00298.x

[B3] Barrera D. J. Rosenberg J. N. Chiu J. G. Chang Y. N. Debatis M. Ngoi S. M. (2015). Algal chloroplast produced camelid VH H antitoxins are capable of neutralizing botulinum neurotoxin. *Plant Biotechnol. J.* 13 117–124. 10.1111/pbi.12244 25229405 PMC4620920

[B4] Bock R. (2007). Plastid biotechnology: prospects for herbicide and insect resistance, metabolic engineering and molecular farming. *Curr. Opin. Biotechnol.* 18 100–106. 10.1016/j.copbio.2006.12.001 17169550

[B5] CDC. (2020). Preliminary incidence and trends of infections with pathogens transmitted commonly through food — foodborne diseases active surveillance network, 10 U.S. Sites, 2016–2019. *Morb. Mortal. Wkly Rep.* 69 509–514. 10.15585/mmwr.mm6917a1 32352955 PMC7206985

[B6] Chin-Fatt A. S. Saberianfar R. Menassa R. (2021). A rationally designed bovine IgA Fc scaffold enhances in planta accumulation of a V_H_H-Fc fusion without compromising binding to enterohemorrhagic E. coli. *Front. Plant Sci.* 12:636. 10.3389/fpls.2021.651262 33936135 PMC8079772

[B7] Daniell H. Lee S. B. Panchal T. Wiebe P. O. (2001). Expression of the native cholera toxin B subunit gene and assembly as functional oligomers in transgenic tobacco chloroplasts. *J. Mol. Biol.* 311 1001–1009. 10.1006/jmbi.2001.4921 11531335 PMC3473180

[B8] Daniell H. Singh N. D. Mason H. Streatfield S. J. (2009). Plant-made vaccine antigens and biopharmaceuticals. *Trends Plant Sci.* 14 669–679. 10.1016/j.tplants.2009.09.009 19836291 PMC2787751

[B9] De Marchis F. Pompa A. Mannucci R. Morosinotto T. Bellucci M. (2011). A plant secretory signal peptide targets plastome-encoded recombinant proteins to the thylakoid membrane. *Plant Mol. Biol.* 76 427–441. 10.1007/s11103-010-9676-6 20714919

[B10] De Meyer T. Muyldermans S. Depicker A. (2014). Nanobody-based products as research and diagnostic tools. *Trends Biotechnol.* 32 263–270. 10.1016/j.tibtech.2014.03.001 24698358

[B11] De Wilde K. De Buck S. Vanneste K. Depicker A. (2013). Recombinant antibody production in arabidopsis seeds triggers an unfolded protein response. *Plant Physiol.* 161 1021–1033. 10.1104/pp.112.209718 23188806 PMC3561000

[B12] Emanuelsson O. Nielsen H. Von Heijne G. (1999). ChloroP, a neural network-based method for predicting chloroplast transit peptides and their cleavage sites. *Protein Sci.* 8 978–984. 10.1110/ps.8.5.978 10338008 PMC2144330

[B13] Fernandez D. E. (2018). Two paths diverged in the stroma: targeting to dual SEC translocase systems in chloroplasts. *Photosynth. Res.* 138 277–287. 10.1007/s11120-018-0541-9 29951837

[B14] Harmsen M. M. De Haard H. J. (2007). Properties, production, and applications of camelid single-domain antibody fragments. *Appl. Microbiol. Biotechnol.* 77 13–22. 10.1007/s00253-007-1142-2 17704915 PMC2039825

[B15] Heineke D. Riens B. Grosse H. Hoferichter P. Peter U. Flügge U. I. (1991). Redox transfer across the inner chloroplast envelope membrane. *Plant Physiol.* 95 1131–1137. 10.1104/pp.95.4.1131 16668101 PMC1077662

[B16] Henry K. A. Kandalaft H. Lowden M. J. Rossotti M. A. Van Faassen H. Hussack G. (2017). A disulfide-stabilized human VL single-domain antibody library is a source of soluble and highly thermostable binders. *Mol. Immunol.* 90 190–196. 10.1016/j.molimm.2017.07.006 28820969

[B17] Herr A. B. Ballister E. R. Bjorkman P. J. (2003). Insights into IgA-mediated immune responses from the crystal structures of human FcαRI and its complex with IgA1-Fc. *Nature* 423 614–620. 10.1038/nature01685 12768205

[B18] Herrmann J. M. Kauff F. Neuhaus H. E. (2009). Thiol oxidation in bacteria, mitochondria and chloroplasts: common principles but three unrelated machineries? *Biochim. Biophys. Acta* 1793 71–77. 10.1016/j.bbamcr.2008.05.001 18522807

[B19] Jobling S. A. Jarman C. Teh M. M. Holmberg N. Blake C. Verhoeyen M. E. (2003). Immunomodulation of enzyme function in plants by single-domain antibody fragments. *Nat. Biotechnol.* 21 77–80. 10.1038/nbt772 12483224

[B20] Johnson M. P. Wientjes E. (2019). The relevance of dynamic thylakoid organisation to photosynthetic regulation. *Biochim. Biophys. Acta Bioenerg.* 1861:148039. 10.1016/j.bbabio.2019.06.011 31228404

[B21] Karamoko M. Gabilly S. T. Hamel P. P. (2013). Operation of trans-thylakoid thiol-metabolizing pathways in photosynthesis. *Front. Plant Sci.* 4:476. 10.3389/fpls.2013.00476 24348486 PMC3842002

[B22] Kieselbach T. Schroder W. P. (2003). The proteome of the chloroplast lumen of higher plants. *Photosynth. Res.* 78 249–264. 10.1023/b:pres.0000006913.86689.f1 16245054

[B23] King M. T. Huh I. Shenai A. Brooks T. M. Brooks C. L. (2018). Structural basis of V_H_H-mediated neutralization of the food-borne pathogen Listeria monocytogenes. *J. Biol. Chem.* 293 13626–13635. 10.1074/jbc.ra118.003888 29976754 PMC6120195

[B24] Kirchhoff H. (2019). Chloroplast ultrastructure in plants. *New Phytol.* 223 565–574. 10.1111/nph.15730 30721547

[B25] Kolotilin I. Kaldis A. Devriendt B. Joensuu J. Cox E. Menassa R. (2012). Production of a subunit vaccine candidate against porcine post-weaning diarrhea in high-biomass transplastomic tobacco. *PLoS One* 7:e42405. 10.1371/journal.pone.0042405 22879967 PMC3411772

[B26] Kubis S. E. Lilley K. S. Jarvis P. (2008). Isolation and preparation of chloroplasts from arabidopsis thaliana plants. *Methods Mol. Biol.* 425 171–186. 10.1007/978-1-60327-210-0_16 18369897

[B27] Kumar N. Arthur C. P. Ciferri C. Matsumoto M. L. (2020). Structure of the secretory immunoglobulin a core. *Science* 367 1008–1014. 10.1126/science.aaz5807 32029686

[B28] Kumar S. Dhingra A. Daniell H. (2004). Stable transformation of the cotton plastid genome and maternal inheritance of transgenes. *Plant Mol. Biol.* 56 203–216. 10.1007/s11103-004-2907-y 15604738 PMC3481848

[B29] Lee D. W. Lee J. Hwang I. J. (2017). Sorting of nuclear-encoded chloroplast membrane proteins. *Curr. Opin. Plant Biol.* 40 1–7. 10.1016/j.pbi.2017.06.011 28668581

[B30] Li M. Z. Elledge S. J. (2007). Harnessing homologous recombination in vitro to generate recombinant DNA via SLIC. *Nat. Methods* 4 251–256. 10.1038/nmeth1010 17293868

[B31] Li Y. Jin L. Chen T. (2020). The effects of secretory IgA in the mucosal immune system. *Biomed. Res. Int.* 2020:2032057. 10.1155/2020/2032057 PMC6970489 31998782

[B32] Lu Y. Wang H.-R. Li H. Cui H.-R. Feng Y.-G. Wang X.-Y. (2013). A chloroplast membrane protein LTO1/AtVKOR involving in redox regulation and ROS homeostasis. *Plant Cell. Rep.* 32 1427–1440. 10.1007/s00299-013-1455-9 23689258

[B33] Ma J. K. C. Hikmat B. Y. Wycoff K. Vine N. D. Chargelegue D. Yu L. (1998). Characterization of a recombinant plant monoclonal secretory antibody and preventive immunotherapy in humans. *Nat. Med.* 4 601–606. 10.1038/nm0598-601 9585235

[B34] Magee A. M. Coyne S. Murphy D. Horvath E. M. Medgyesy P. Kavanagh T. A. (2004). T7 RNA polymerase-directed expression of an antibody fragment transgene in plastids causes a semi-lethal pale-green seedling phenotype. *Transgenic Res.* 13 325–337. 10.1023/b:trag.0000040019.35147.a4 15517992

[B35] Mayfield S. P. Franklin S. E. Lerner R. A. (2003). Expression and assembly of a fully active antibody in algae. *Proc. Natl. Acad. Sci. U S A* 100 438–442. 10.1073/pnas.0237108100 12522151 PMC141013

[B36] Nakanishi K. Narimatsu S. Ichikawa S. Tobisawa Y. Kurohane K. Niwa Y. (2013). Production of hybrid-IgG/IgA plantibodies with neutralizing activity against Shiga toxin 1. *PLoS One* 8:e80712. 10.1371/journal.pone.0080712 24312238 PMC3842918

[B37] Onda Y. (2013). Oxidative protein-folding systems in plant cells. *Int. J. Cell Biol.* 2013:585431. 10.1155/2013/585431 PMC3800646 24187554

[B38] Palmer T. Stansfeld P. (2020). Targeting of proteins to the twin-arginine translocation pathway. *Mol. Microbiol.* 113 861–871. 10.1111/mmi.14461 31971282 PMC7317946

[B39] Pereira E. O. Kolotilin I. Conley A. J. Menassa R. (2014). Production and characterization of in planta transiently produced polygalacturanase from Aspergillus niger and its fusions with hydrophobin or ELP tags. *BMC Biotechnol* 14:59. 10.1186/1472-6750-14-59 24970673 PMC4083859

[B40] PHAC. (2019). *FoodNet Canada Annual Report 2018.* Available Online at: https://www.canada.ca/en/public-health/services/surveillance/foodnet-canada/publications/foodnet-canada-annual-report-2018.html Accessed 21 Mar 2021

[B41] Pottosin I. Shabala S. (2016). Transport across chloroplast membranes: optimizing photosynthesis for adverse environmental conditions. *Mol. Plant* 9 356–370. 10.1016/j.molp.2015.10.006 26597501

[B42] Ramsland P. A. Willoughby N. Trist H. M. Farrugia W. Hogarth P. M. Fraser J. D. (2007). Structural basis for evasion of IgA immunity by *Staphylococcus aureus* revealed in the complex of SSL7 with Fc of human IgA1. *Proc. Nat. Acad. Sci.* 104 15051–15056. 10.1073/pnas.0706028104 17848512 PMC1986611

[B43] Ries F. Herkt C. Willmund F. (2020). Co-translational protein folding and sorting in chloroplasts. *Plants* 9:214. 10.3390/plants9020214 32045984 PMC7076657

[B44] Robinson C. Matos C. F. Beck D. Ren C. Lawrence J. Vasisht N. (2011). Transport and proofreading of proteins by the twin-arginine translocation (Tat) system in bacteria. *Biochim. Biophys. Acta* 1808 876–884. 10.1016/j.bbamem.2010.11.023 21126506

[B45] Saberianfar R. Chin-Fatt A. Scott A. Henry K. A. Topp E. Menassa R. (2019). Plant-produced chimeric V_H_H-sIgA against enterohemorrhagic E. coli Intimin shows cross-serotype inhibition of bacterial adhesion to epithelial cells. *Front. Plant Sci.* 10:270. 10.3389/fpls.2019.00270 30972081 PMC6445026

[B46] Schlicher T. Soll J. (1996). Molecular chaperones are present in the thylakoid lumen of pea chloroplasts. *FEBS Lett.* 379 302–304. 10.1016/0014-5793(95)01534-5 8603711

[B47] Schmidt D. J. Beamer G. Tremblay J. M. Steele J. A. Kim H. B. Wang Y. (2016). A tetraspecific V_H_H-based neutralizing antibody modifies disease outcome in three animal models of clostridium difficile infection. *Clin. Vaccine Immunol.* 23 774–784. 10.1128/cvi.00730-15 27413067 PMC5014919

[B48] Schubert M. Petersson U. A. Haas B. J. Funk C. Schroder W. P. Kieselbach T. (2002). Proteome map of the chloroplast lumen of arabidopsis thaliana. *J. Biol. Chem.* 277 8354–8365. 10.1074/jbc.m108575200 11719511

[B49] Schürmann P. Buchanan B. B. (2008). The ferredoxin/thioredoxin system of oxygenic photosynthesis. *Antioxid Redox Signal* 10 1235–1274. 10.1089/ars.2007.1931 18377232

[B50] Shkoporov A. Khokhlova E. Savochkin K. Kafarskaia L. Efimov B. (2015). Production of biologically active scFv and V_H_H antibody fragments in bifidobacterium longum. *FEMS Microbiol. Lett.* 362:fnv083. 10.1093/femsle/fnv083 25994292

[B51] Silhavy D. Molnar A. Lucioli A. Szittya G. Hornyik C. Tavazza M. (2002). A viral protein suppresses RNA silencing and binds silencing-generated, 21- to 25-nucleotide double-stranded RNAs. *Embo J.* 21 3070–3080. 10.1093/emboj/cdf312 12065420 PMC125389

[B52] Staub J. M. Garcia B. Graves J. Hajdukiewicz P. T. Hunter P. Nehra N. (2000). High-yield production of a human therapeutic protein in tobacco chloroplasts. *Nat. Biotechnol.* 18 333–338. 10.1038/73796 10700152

[B53] Takizawa K. Cruz J. A. Kanazawa A. Kramer D. M. (2007). The thylakoid proton motive force in vivo. Quantitative, non-invasive probes, energetics, and regulatory consequences of light-induced pmf. *Biochim. Biophys. Acta* 1767 1233–1244. 10.1016/j.bbabio.2007.07.006 17765199

[B54] Tissot G. Canard H. Nadai M. Martone A. Botterman J. Dubald M. (2008). Translocation of aprotinin, a therapeutic protease inhibitor, into the thylakoid lumen of genetically engineered tobacco chloroplasts. *Plant Biotechnol. J.* 6 309–320. 10.1111/j.1467-7652.2008.00321.x 18266824

[B55] Trebitsh T. Meiri E. Ostersetzer O. Adam Z. Danon A. (2001). The protein disulfide isomerase-like RB60 is partitioned between stroma and thylakoids in chlamydomonas reinhardtii chloroplasts. *J. Biol. Chem.* 276 4564–4569. 10.1074/jbc.m005950200 11087734

[B56] Vasilev N. Smales C. M. Schillberg S. Fischer R. Schiermeyer A. (2016). Developments in the production of mucosal antibodies in plants. *Biotechnol. Adv.* 34 77–87. 10.1016/j.biotechadv.2015.11.002 26626615

[B57] Vega C. G. Bok M. Vlasova A. N. Chattha K. S. Gómez-Sebastián S. Nuñez C. (2013). Recombinant monovalent llama-derived antibody fragments (V_H_H) to rotavirus VP6 protect neonatal gnotobiotic piglets against human rotavirus-induced diarrhea. *PLoS Pathog.* 9:e1003334. 10.1371/journal.ppat.1003334 23658521 PMC3642062

[B58] Vincke C. Muyldermans S. (2012). Introduction to heavy chain antibodies and derived nanobodies. *Methods Mol. Biol.* 911 15–26. 10.1007/978-1-61779-968-6_2 22886243

[B59] Virdi V. Coddens A. De Buck S. Millet S. Goddeeris B. M. Cox E. (2013). Orally fed seeds producing designer IgAs protect weaned piglets against enterotoxigenic *Escherichia coli* infection. *Proc. Natl. Acad. Sci. U S A* 110 11809–11814. 10.1073/pnas.1301975110 23801763 PMC3718133

[B60] Virdi V. Palaci J. Laukens B. Ryckaert S. Cox E. Vanderbeke E. (2019). Yeast-secreted, dried and food-admixed monomeric IgA prevents gastrointestinal infection in a piglet model. *Nat. Biotechnol.* 37 527–530. 10.1038/s41587-019-0070-x 30936561 PMC6544532

[B61] Werdan K. Heldt H. W. Milovancev M. (1975). The role of pH in the regulation of carbon fixation in the chloroplast stroma. Studies on CO2 fixation in the light and dark. *Biochim. Biophys. Acta.* 396 276–292. 10.1016/0005-2728(75)90041-9 239746

[B62] Wesolowski J. Alzogaray V. Reyelt J. Unger M. Juarez K. Urrutia M. (2009). Single domain antibodies: promising experimental and therapeutic tools in infection and immunity. *Med. Microbiol. Immunol.* 198 157–174. 10.1007/s00430-009-0116-7 19529959 PMC2714450

[B63] Woof J. Russell M. (2011). Structure and function relationships in IgA. *Mucosal. Immunol.* 4 590–597. 10.1038/mi.2011.39 21937984

[B64] Xiong Y. Karuppanan K. Bernardi A. Li Q. Kommineni V. Dandekar A. M. (2019). Effects of N-glycosylation on the structure, function, and stability of a plant-made fc-fusion anthrax decoy protein. *Front. Plant Sci.* 10:768. 10.3389/fpls.2019.00768 31316527 PMC6611495

